# Poor man’s fluorescence?

**DOI:** 10.1007/s00701-015-2471-z

**Published:** 2015-06-19

**Authors:** Walter Stummer

**Affiliations:** Department of Neurosurgery, University Hospital Münster, Albert-Schweitzer-Campus 1, Gebäude A1, 48149 Münster, Germany

Dear Editor,

We thank you for the opportunity for commenting on the letter by Ascerbi and co-workers. We feel the discussions surrounding fluorescence stimulating and interesting.

Our first experience with fluorescein was in 1991 when we used fluorescein for marking perilesional edema in the cortical cold lesion model [21]. We demonstrated fluorescein propagating through the brain, driven by edema in the absence of tumor. Thus, to us fluorescein has always been an excellent edema marker.

Similarly, when first using fluorescein for brain tumor surgery Moore et al. (1948) [17] noted that “edematous brain surrounding the tumor does fluoresce, but to a lesser degree …”

Since then, investigators have taken up this technology on and off, e.g., Kuoriwa and co-workers [11–13] for brain tumor surgery. This group, incidentally, gave up fluorescein in 1999, later pursuing ALA intensely [6–10, 15, 16, 22–24].

Currently, due to the availability of new filter systems, investigators have again adopted fluorescein for malignant gliomas. Anybody interested in the present use of this technique should appraise footage from fluorescein surgery on the internet (e.g., https://www.youtube.com/watch?v=mST6oPE69dE and http://abcnews.go.com/Health/making-brain-tumors-glow-saves-lives/story?id=17076243#) and draw their own conclusions.

Nonetheless, filters will not change biology and fluorescein will still only mark areas of the brain with blood–brain barrier breakdown, which are somewhat but not strictly related to tumor. Novel experimental work has confirmed fluorescein staining to not be tumor-cell specific [5]. Extravasation and propagation of fluorescein with edema will follow a distinctive time course: after administration, levels will be high in blood and perfused tissues, blood and CSF. With a half-life of 264 min, intravascular fluorescein will slowly subside, in the mean time being extravasated with edema and travelling through peritumoral tissue, raising the danger of staining non-tumorous brain. Thus, the timing of surgery will be critical. The second problem arises from surgical tissue injury. Any injury to the tissue will result in extravasation of fluorescein at the cut margin and any other areas of tissue injury. Fluorescein in blood will stain such injured tissues. None of the authors currently reporting on fluorescein discuss this much, although these phenomena are clearly visible in images from their publications (e.g., Fig. 1 in Ascerbi et al. 2014 [1]; Fig. 3e, f in Li et al. 2014 [14]; Fig. 3b in Schebesch et al. 2013 [20]; Fig. 1e in Rey-Dios and Cohen-Gadol et al. (2013) [19]; Fig. 7c in Diaz et al. 2015 [5]; Fig. 4b in Okuda et al. (2012) [18]), where parts of the margin of the cavity and/or the surrounding cortical tissues are stained after completion of tumor removal. Why didn’t the authors continue resection in these areas with clearly visible fluorescence?

Together, timing and dose appear to be essential, as Ascerbi et al. acknowledge in their letter, but there does not appear to be much consensus among current investigators regarding these two aspects (Table 1). Ascerbi et al. suggest 5 mg/kg to be administered i.v. after intubation, i.e., approximately 1 h prior to dural opening. What happens when craniotomies take longer or the tumor is large or when craniotomies are fast and the tumors small? In no publication so far is the temporal aspect accounted for when regarding fluorescence in the later stages of resection and its influence on diagnostic accuracy.

**Table 1:** When and how should fluorescein be injected (publications after 2012)

Publication	Information on biopsies for determining diagnostic accuracy (e.g., how many per patient, exact location)	Information on timing of biopsy after administration	Timing of injection and dose
Rey-Dios and Cohen-Gadol 2013 [19]	No biopsies	No biopsies	15 min before dural opening, 4 mg/kg fluorescein
Schebesch et al. 2013 [20]	No biopsies	No biopsies	200 mg corresponding to approximately 3–4 mg/kg) of fluorescein were intravenously administered after bone flap removal prior to durotomy
Ascerbi et al. 2014 [1], Ascerbi et al. 2013 [2]	“Four biopsy specimens were obtained at the tumor margin in areas located distant from each other—2 in the fluorescent tissue and 2 in the nonfluorescent tissue”	No	5–10 mg/kg intravenously “after intubation and before skin incision,” BLUE 400 and Yellow 560 filters
Chen et al. 2012 [4]	No biopsies	No biopsies	“After the dura at the craniotomy site was opened, fluorescein sodium (diluted into 1 %, 5 ml; Alcon, USA, 5 ml: 500 mg, import drug ID was H20090507) was injected intravenously. Vital signs were monitored for 15 min. Following confirmation that no skin rash was present, 10 ml of high-dose fluorescein sodium (diluted into 10 %, 15–20 mg/kg)”
Diaz et al. 2015 [5]	“… multiple (3–8) random fluorescent and non-fluorescent samples were obtained at the tumor margins defined by fluorescence and MR neuronavigation … the nonfluorescent or minimally fluorescent margins after resection were also biopsied … and … labeled “non-fluorescent”	No	3 mg/kg bolus at the time of anesthesia induction
Okuda et al. 2012 [18]	No biopsies	No biopsies	20 mg/kg after induction of anesthesia and opening of the dura

What we are simply saying here is that fluorescein is not well investigated at this point and harbors a number of pitfalls and hazards to justify too much enthusiasm. Frankly, surgeons should be wary.

Surgical studies are tricky and the possibilities for advertent or inadvertent bias numerous. Especially in studies on intraoperative optical diagnosis, calculations of specificity and sensitivity are highly influenced by sample location and number of samples per patient. Thus, a high level of transparency and control are necessary for such investigations, including exact and objective description of how many biopsies were taken, exactly from where and how a per-patient analysis compared with a per-biopsy analysis (problematical mixing of dependent and independent data) are handled. In addition, publications should strictly adhere to the STARD statement [3] for studies on diagnostic accuracy. It is not apparent that this is happening to a satisfactory extent in available reports on the clinical use of fluorescein (Table 1). Clinical studies within a proper GCP setting, including pre-defined study aims, biometry and external monitoring would be more than welcome at this time.

Finally, the argument is frequently put forward [14, 20] that fluorescein is cheaper than ALA and thus better. This perception is worrisome. If I were a patient with a brain tumor, I would want something that is better or equally good. The price would not be my worry.
